# Supplementary material for: Neural crest cell biology shapes lizard skull evolution across evolutionary time scales
Source: Evol Lett. 2025 Dec 26;10(2):152–64. doi: 10.1093/evlett/qraf050 (PMC13043907; doi:10.1093/evlett/qraf050)
Supplement: qraf050_Supplemental_File [file qraf050_supplemental_file.pdf]

Neural crest cell biology shapes lizard skull evolution across evolutionary time scales

Supplementary Figures and Tables

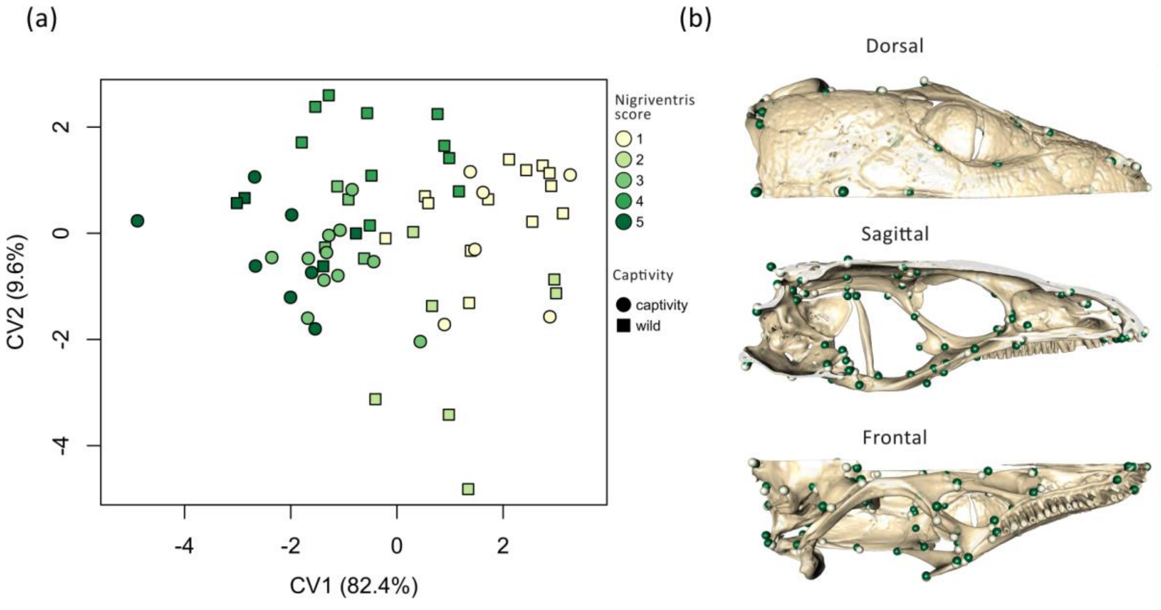

Fig. S1. Canonical variate analysis of landmark configurations on the skull of *Podarcis muralis* with raw data instead of size-corrected residuals. Nigriventris score categories range from populations with ancestral like phenotype to populations with the most extreme expressions of the nigriventris syndrome. (a) Projection of the first two canonical variates (CV), (b) landmark displacement between the two extremes of CV1 (min: light green; max: dark green). Inverse of CV1 displayed for comparisons with Figure 2.

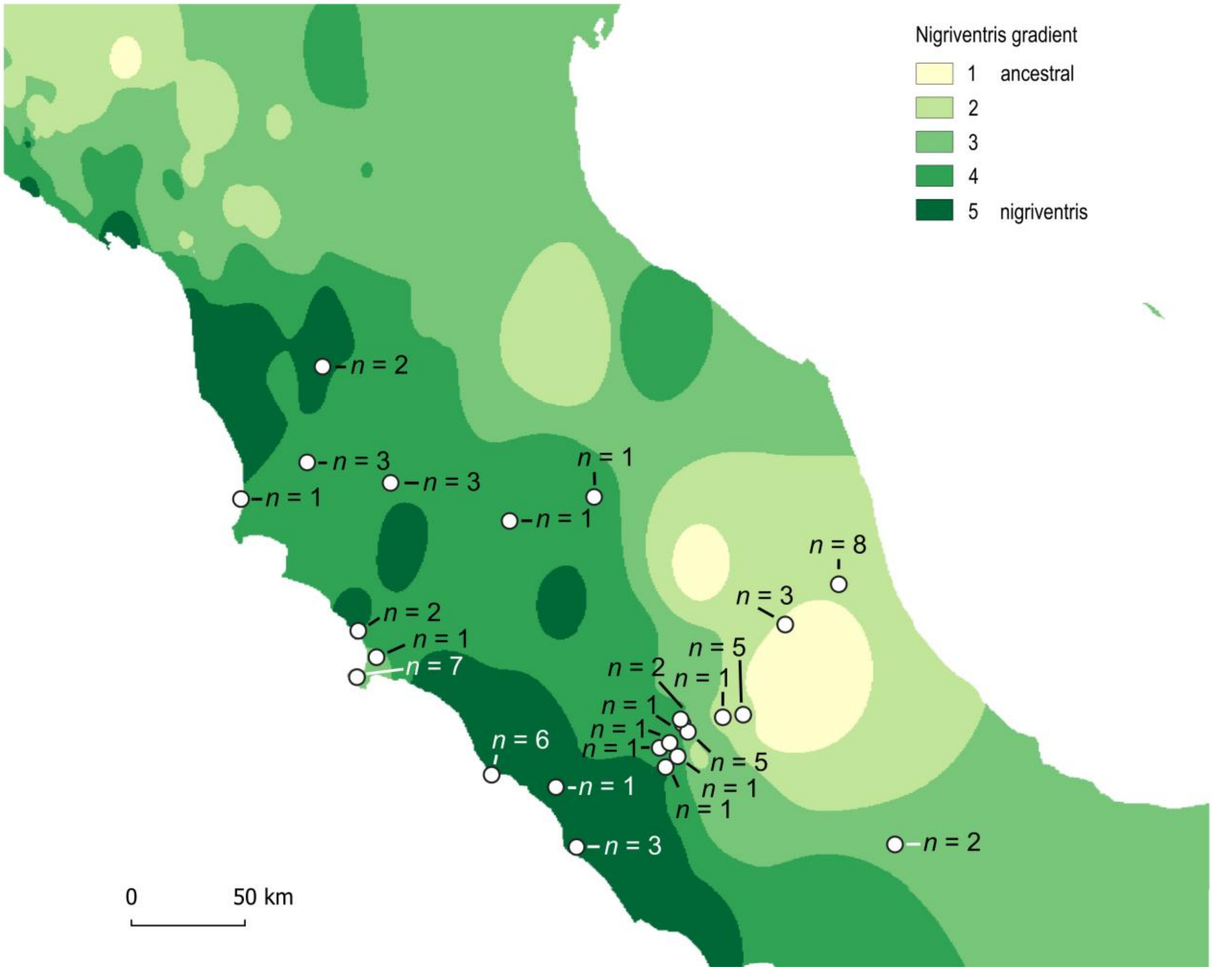

Fig. S2. Locations and sample sizes of *Podarcis muralis* specimens from central Italy used for the study. Distribution of dorsal green coloration, a proxy of the strength of the nigriventris syndrome, from ancestral type to extreme nigriventris phenotype, is based on the estimates from Ruiz Miñano *et al.* (2021) and Feiner *et al.* (2024). The dorsal green colour variations distribution is coarsely extrapolated for illustrative purpose, the final decisions to attribute nigriventris scores to the populations were made based on thorough considerations of the fine scale data from Ruiz Miñano *et al.* (2021) and Feiner *et al.* (2024).

Table S1. Posterior estimates of head length (linear measurements) in *P. muralis* from a linear model (MCMC GLMM) including nigriventris score as a fixed effect [1].

Effect	Posterior mode	95% CrI	Effective sample size	pMCMC
Origin - captivity	0.271	-0.066: 0.63	1000	0.13
Origin - field	-0.26	-0.439: -0.098	1000	0.004
nigriventris score 2	0.169	-0.835: 1.277	1000	0.636
nigriventris score 3	0.713	-0.644: 1.498	1000	0.482
nigriventris score 4	0.336	-0.233: 0.906	1251	0.334
nigriventris score 5	0.337	**0.023: 0.467**	1000	**0.016**
Body length (SVL)	0.245	0.198: 0.268	1000	0.001
nigriventris score 2 x SVL	-0.059	-0.187: 0.039	1000	0.228
nigriventris score 3 x SVL	0.103	-0.087: 0.226	1000	0.296
nigriventris score 4 x SVL	0.027	-0.057: 0.095	1000	0.594
nigriventris score 5 x SVL	0.021	-0.017: 0.068	1000	0.302

[1]Formula: head length ~ captivity + nigriventris score x body length – 1. Iterations: 130,000; burnins: 30,000; thinning intervals: 100. Baseline: nigriventris score 1.

Table S2. Pairwise comparison of the allometric slope of nigriventris categories in *P. muralis*.

	**Procrustes distances**			**Vector correlations**			
**Pairs**	***d***	**Z**	$\boldsymbol{P}_{\mathbf{distance}}$	***r***	**angle**	**Z**	$\boldsymbol{P}_{\mathbf{angle}}$
1 vs. 2	0.022	**1.810**	**0.036**	-0.15	1.721	**3.546**	**<0.001**
1 vs. 3	0.016	**2.171**	**0.014**	0.281	1.286	**2.639**	**0.004**
1 vs. 4	0.013	0.320	0.374	0.264	1.303	**3.328**	**<0.001**
1 vs. 5	0.019	**2.042**	**0.019**	0.46	1.092	0.902	0.184
2 vs. 3	0.021	0.865	0.192	0.375	1.186	0.276	0.39
2 vs. 4	0.021	1.476	0.068	0.519	1.025	-0.794	0.789
2 vs. 5	0.022	0.488	0.315	0.169	1.401	1.498	0.066
3 vs. 4	0.014	-0.992	0.840	0.728	0.755	-0.735	0.768
3 vs. 5	0.016	0.862	0.195	0.485	1.064	0.768	0.221
4 vs. 5	0.016	-0.788	0.786	0.56	0.977	0.684	0.244

References

Feiner, N., Yang, W., Bunikis, I., While, G.M. & Uller, T. (2024) Adaptive introgression reveals the genetic basis of a sexually selected syndrome in wall lizards. *Science Advances*, **10**.

Ruiz Miñano, M., While, G.M., Yang, W., Burridge, C.P., Sacchi, R., Zuffi, M., *et al.* (2021) Climate Shapes the Geographic Distribution and Introgressive Spread of Color Ornamentation in Common Wall Lizards. *The American Naturalist*, **198**, 379–393.
